# A Roadmap for Genome-Based Phage Taxonomy

**DOI:** 10.3390/v13030506

**Published:** 2021-03-18

**Authors:** Dann Turner, Andrew M. Kropinski, Evelien M. Adriaenssens

**Affiliations:** 1 Department of Applied Sciences, University of the West of England, Bristol BS16 1QY, UK; dann2.turner@uwe.ac.uk; 2 Department of Food Science, University of Guelph, Guelph, ON N1G 2W1, Canada; phage.canada@gmail.com; 3 Department of Pathobiology, University of Guelph, Guelph, ON N1G 2W1, Canada; 4 Quadram Institute Bioscience, Norwich Research Park, Norwich NR4 7UQ, UK

**Keywords:** phage taxonomy, phage classification, *Caudovirales*, *Myoviridae*, *Podoviridae*, *Siphoviridae*, demarcation criteria

## Abstract

Bacteriophage (phage) taxonomy has been in flux since its inception over four decades ago. Genome sequencing has put pressure on the classification system and recent years have seen significant changes to phage taxonomy. Here, we reflect on the state of phage taxonomy and provide a roadmap for the future, including the abolition of the order *Caudovirales* and the families *Myoviridae*, *Podoviridae,* and *Siphoviridae*. Furthermore, we specify guidelines for the demarcation of species, genus, subfamily and family-level ranks of tailed phage taxonomy.

## 1. An Ongoing Revolution in Phage Taxonomy

**Figure 1:**
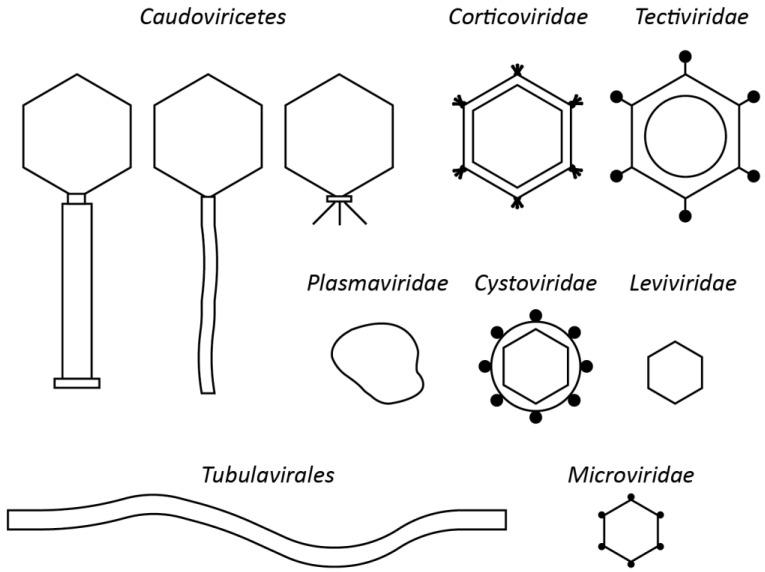
Line drawing of bacteriophage morphotypes, adapted from Ackermann, 2005 [6].

Historically, phages have been classified according to their morphology, dating from the time before the existence of PCR, sequencing or many of the molecular methods we know today [1,2,3]. For tailed phages, the formal taxonomy was derived from the pioneering classification work of David Bradley (Memorial University, Canada) who classified them into three morphotypes, A (contractile tail), B (long, non-contractile tail), C (short non-contractile tail, based on electron microscopy, a system that was subsequently enhanced by Ackermann and Eisenstark (1974) [4,5]. In 1971, this system was formally adopted by the International Committee on Nomenclature of Viruses (ICNV) but not with the names we are familiar with today. The names *Myoviridae*, *Podoviridae* and *Siphoviridae* were formally accepted by the International Committee on Taxonomy of Viruses (ICTV) in 1981 and 1984. The order *Caudovirales*, unifying all tailed phages, was proposed in 1998 by Hans-Wolfgang Ackermann and approved by postal vote. Some of the other phage families have equally long histories with the families *Inoviridae*, *Microviridae, Tectiviridae*, *Corticoviridae*, *Plasmaviridae*, *Leviviridae,* and *Cystoviridae* all formalised by plenary session vote in 1978 (for a history of taxonomy releases see https://talk.ictvonline.org/taxonomy/p/taxonomy_releases, accessed on 5 February 2021). This >40-year-old family-level classification system resulted in the classic textbook figures (Figure 1) on phage taxonomy, easily represented by line drawings.

As the age of genomics dawned in the early 2000s, the sequencing of phage genomes revealed a much higher genomic diversity than had previously been considered, especially in bacteriophages belonging to the order *Caudovirales*, leading to the creation of the first subfamilies within the existing three families *Podoviridae* [7], *Myoviridae* [8], and later on *Siphoviridae* [9]. As the number of phage genomes in databases rose, it quickly became apparent that these three families were not monophyletic and cohesive within a monophyletic order. This paraphyly was illustrated by a number of tools and publications: The Phage Proteomic Tree [10,11], the first phage genome relatedness network representation [12], a bipartite network of shared genes [13], an updated network of shared predicted proteins (vConTACT) [14,15], a composite tool combining gene homologies and gene order (GRAViTy) [16,17], a virus domain orthologous groups approach (VDOG) [18] and a concatenated protein phylogeny of members of the order *Caudovirales* (CCP77) [19]. Based on this evidence, the ICTV’s Bacterial and Archaeal Viruses Subcommittee started disentangling the web of overlapping and complementary groups of tailed phages by defining new, genome-based families. At the time of writing, three new families of myoviruses have been officially ratified *Ackermannviridae* [20], *Chaseviridae* [21], *Herelleviridae* [22,23]; two for the siphoviruses, *Demerecviridae* [21], and *Drexlerviridae* [21], and one of podoviruses, *Autographiviridae* [21]. 

If we look beyond the traditional tailed bacteriophages, we are observing a similar increase in genomic diversity in other phage clades, but interestingly, these expansions are mainly driven by metagenome-derived information. Using a combination of sequencing, isolation and imaging methods, a new major lineage of non-tailed dsDNA phages was identified in marine bacteria, named *Autolykiviridae* [24]. Similarly, isolation of a new ssDNA phage and description of the new family *Finnlakeviridae* links non-tailed icosahedral ssDNA and dsDNA phages together [25,26]. Major lineages of presumed novel dsDNA tailed phages have also been inferred and isolated based on metagenomic/viromic assemblies, including the crAssphage lineage [27,28,29], Lak megaphage [30], and multiple other lineages of “huge phages” [31]. 

For the filamentous, ssDNA phages, the family *Inoviridae* has been split into two families, *Inoviridae* and *Plectroviridae* which are grouped together in the order *Tubulavirales* [21], with a potential further increase with five new families based on the analysis of cryptic inoviruses from bacterial genome datasets [32]. In a similar vein, many additional subfamilies have been proposed in the ssDNA family *Microviridae*, beyond the existing subfamilies *Bullavirinae* [9] and *Gokushovirinae* based on the detection in virome data, i.e. the subfamilies “Alpavirinae” [33], “Pichovirinae” [34], “Stokavirinae” [35], and “Aravirinae” [35]. Recently, computational approaches identified a massive expansion in the number of ssRNA phage genomes of the *Leviviridae* family, first with 158 [36] then with a further 1k complete and 15k partial genomes [37]. 

Across all the different lineages of bacteriophages it has become clear that fundamental changes to classification are required in order to address this increasing genomic diversity.

## 2. The Next Steps for Tailed Phage Taxonomy

**Figure 2:**
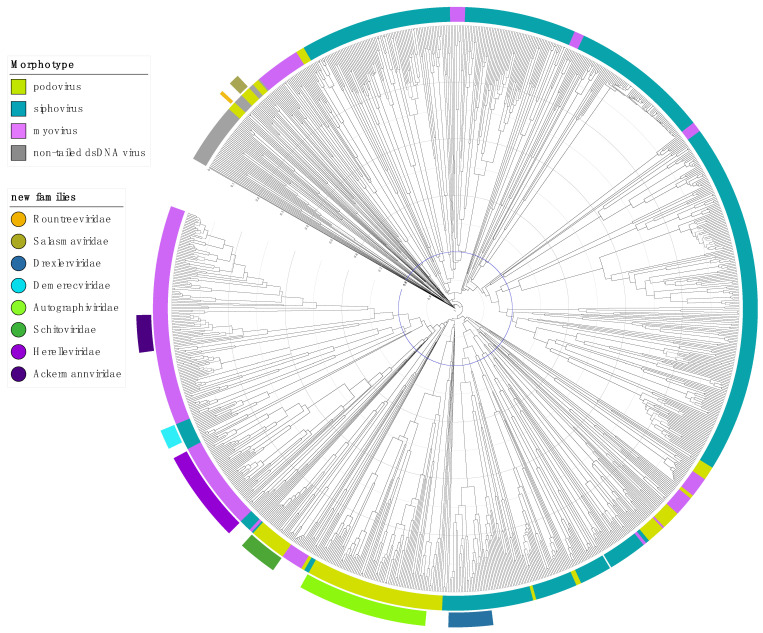
Dendrogram generated by GRAViTy (http://gravity.cvr.gla.ac.uk, accessed on 5 February 2021) for DB-B: Baltimore Group Ib—Prokaryotic and archaeal dsDNA viruses (VMRv34) and annotated using iTOL [16,42]. The inside coloured ring indicates the morphotype and the outside ring the new proposed and ratified families as of 2021. The distance from tip to node, indicated by the scale rings, represents the composite generalised Jaccard distance (0–1) between two genomes calculated based on relatedness of the proteins and the genome organisation, where 0 is identical and 1 is no measurable relation between two genomes. The Jaccard distance of 0.8, unifying the majority of eukaryotic virus families is indicated in blue for illustration purposes. Bootstrap values (0–1) are indicated by branch colour on a greyscale, from light grey (0) to black (1), showing that the majority of branches are well-supported. Bootstrap values were calculated as described by Aiewsakun and Simmonds [16] by random resampling of the protein profile hidden Markov models that form the basis of the protein relatedness score, recomputing the pairwise distance matrix and then recomputing the dendrogram and repeating this 100 times.

Within phage taxonomy, the most pressing issue remains the paraphyly of the tailed phage families, because they make up the majority of isolated and metagenomically-inferred viruses, illustrated by a dendrogram of hierarchical relations of dsDNA bacterial and archaeal viruses generated by the GRAViTy pipeline (Figure 2, Figure S1) [16,17] and a network-based representation of shared genes generated by vConTACT2 (Figure S2) [15]. 

In recent years, the ICTV expanded the taxonomic ranks, previously Species to Order, to include 15 divisions up to Realm to assist with describing higher order relationships between groups of viruses [38,39]. For the tailed phages, this has led to the introduction of the class *Caudoviricetes* comprising all tailed phages. With the creation of the class, we are now able to abolish the order *Caudovirales* and the families *Myoviridae*, *Podoviridae,* and *Siphoviridae*, and replace them with monophyletic, genome-based families. We have used the creation of the family *Herelleviridae* as a case study for the delineation and internal structuring of future new families [22] but have not addressed the wider implications for all tailed phages. 

### 2.1. Step 1: Abolish the Order Caudovirales

As a first step we propose to abolish the order *Caudovirales* with all current members automatically assigned to the class *Caudoviricetes*. This creates the space to define new orders that group families based on underlying evolutionary relationships. A first example of this is the creation of the order “Crassvirales”, currently under consideration by the ICTV, which groups six families of crAss-like viruses (Taxonomy Proposal 2020.039B, under consideration).

### 2.2. Step 2: Abolish the Families Myoviridae, Podoviridae and Siphoviridae

The removal of the classical phage families will in the first instance create a large number of “unclassified *Caudoviricetes*” subfamilies and genera. While this is a situation that is unsustainable in the long term, in the short term, little taxonomically important information will be lost. For example, the genera *Lederbergvirus* and *Myxoctovirus* are both assigned to the family *Podoviridae,* but their members share no orthologues (verified by CoreGenes 5.0 (coregenes.ngrok.io, accessed on 5 February 2021) as in [40](CoreGenes 3.5 [41]). Therefore, their position as floating genera in the class *Caudoviricetes*, is a better representation of their genomic relatedness than grouping them together in the family *Podoviridae*. 

We do not suggest that the terms myovirus, podovirus, and siphovirus indicating the phage morphology get lost and suggest to use this terminology in publications and add this description in the annotated sequence records (e.g., note in the GenBank file of the genome).

### 2.3. Step 3: Elevating Existing Subfamilies to Family Rank

In the last decade, subfamilies have been created to account for monophyletic groups within the paraphyletic families. For example, the subfamily *Tunavirinae* has been used to create the new family *Drexlerviridae* and the subfamily *Spounavirinae* was the inspiration to create the family *Herelleviridae*. Going forward, there are a number of existing subfamilies such as the *Tevenvirinae* and *Peduovirinae* that are currently being considered for family status, given that their diversity is similar to those of the newly instated families. However, the elevation of subfamilies to families will be assessed on a case-by-case basis. 

### 2.4. Step 4: Addressing the Unclassifieds

While tailed phages exhibit huge genomic diversity, removing the traditional order and families will leave a significant fraction of sequenced phages unclassified at the family-level. In a first instance, we will create floating genera in the class *Caudoviricetes* for these isolates. We propose that for under-represented groups, families should only be created if a sufficient number of genomes, representing multiple genera, have been sequenced to allow the proposal/satisfaction of family-level demarcation criteria (discussed below).

## 3. Rank-Specific Demarcation Criteria for Tailed Phages, Class *Caudoviricetes*

### 3.1. Species

Two phages are assigned to the same species if their genomes are more than 95% identical at the nucleotide level over their full genome length, tested reciprocally. These values can be calculated by a number of tools, such as BLASTn (% identity multiplied by % coverage) [43], VIRIDIC (intergenomic distance calculator, [44]), or CD-HIT-EST [45]. This threshold was first introduced in taxonomy proposals in 2012 [46] and has since been independently confirmed using global population-level analyses [47,48,49]. 

In order to scale up these calculations for the exceedingly large numbers of genomes that are available through metagenomics studies, future studies will need to look into more high-throughput calculations using, for example, genome distance estimations using Mash [50] and appropriate thresholds determined. 

### 3.2. Genus

In search for criteria that create cohesive and distinct genera that are reproducible and monophyletic, the Subcommittee has established 70% nucleotide identity of the full genome length as the cut-off for genera, calculated in the same way as the species cut-off. Pairwise genome comparisons can result in “edge-cases” where inclusion in the genus is only partially supported, needing additional evidence in support. Genomes comprising a proposed genus should be examined for the presence of homologous conserved ‘signature genes’ and evaluated using phylogenetics. 

Various tools have been developed for the assessment of pangenomes (identification of entire gene set of a group of organisms) and, while predominantly designed for the analysis of bacteria, can be employed for the assessment of phage gene products. Examples include Roary [51], Proteinortho [52], PIRATE [53], GET_HOMOLOGUES [54] and CoreGenes 3.5 [41] and 5.0 (https://coregenes.ngrok.io/, accessed on 5 February 2021). We recommend less stringent criteria for the generation of phage pangenomes where sequence similarity and sequence coverage of the proteins are set to >30% identity and >50% coverage, respectively. These approaches allow for hierarchical clustering of phages based on their gene content and demonstrate the presence of signature genes which are stable throughout the genus, subfamily or family. We do encourage phage biologists to check the results of clustering by using multiple sequence alignments and through the use of domain searches (e.g., InterProScan/Pfam/CDD) and more sensitive HMM methods such as hmmscan against the VOGdb and HHPred [55,56,57,58,59]. 

Genus-level groupings should always be monophyletic in these signature genes, as tested by phylogenetic analysis, i.e. the gene or genes chosen as signature(s) for this genus should produce a phylogenetic tree in which the genus is presented as a well-supported single clade. Ideally, phylogenetic trees of signature genes should be rooted using a more distant relative (outgroup) and be accompanied by bootstrap values, to ensure the groupings are robustly reproducible. The Subcommittee recommends Maximum Likelihood (ML) trees built with IQ-Tree, using ModelFinder for substitution model determination and UFBOOT for bootstrapping [60,61,62], but other equivalent tools are acceptable and the Subcommittee has made ample use of the quick and accessible phylogeny.fr webserver for ML-based phylogenetics [63].

### 3.3. Subfamily

The subfamily level is optional for bacteriophages. Subfamilies are to be created when two or more discrete genera are related below the family level. In practical terms, this usually means that they share a low degree of nucleotide sequence similarity and that the genera form a clade in a marker tree phylogeny. 

### 3.4. Family

The family-level has not had any fixed demarcation criteria in the past. Here, we propose the following criteria for the establishment of a new family:

- The family is represented by a cohesive and monophyletic group in the main predicted proteome-based clustering tools (ViPTree, GRAViTy dendrogram, vConTACT2 network).
- Members of the family share a significant number of orthologous genes (the number will depend on the genome sizes and number of coding sequences of members of the family), see genus section for methods.
- If a family-level cluster shares orthologues with another family-level cluster, the family cluster needs to be monophyletic in a phylogenetic analysis of the shared orthologue(s).

### 3.5. Order

Orders should be proposed when two or more families are related. The proposed order should again be monophyletic using the main clustering tools. 

## 4. Perspectives for Non-Tailed Phages

Phages come in a wide variety of genome sizes and compositions. The criteria set out here cannot necessarily be translated for, for example, the small ssRNA genomes of leviviruses, for which a separate set of demarcation criteria are being implemented [37] or the non-tailed dsDNA autolykiviruses [24]. For each of these major groups, new genome-based criteria will need to be developed by groups of experts, but the expectation is that these are broadly equivalent across the bacterial virosphere. We welcome studies that investigate cross-Realm rates of evolution and divergence. 

## 5. Concluding Statement

The classical morphotype family-level taxonomy has been enormously useful for four decades in advancing our understanding of phage diversity. We express our extreme gratitude to those that developed it, in particular the late Hans-Wolfgang Ackermann, who was a supportive yet highly critical collaborator of the authors. For those concerned, while the morphology-based families will disappear, the morphotypes will continue to exist and descriptors such as myovirus and podophage will always remain useful. 

Driven by the renewed interest in phage-based applications, advances in sequencing technology, and the era of the microbiome, there is a dire need for a genome-based classification in which the family level represents a genomic unit of diversity. The first steps on the route towards a future-proof taxonomy have been taken. Here we have laid out our future plans to address the need for a stable and informed taxonomic approach to the viruses of bacteria (and archaea). Implementation of these plans will require the engagement of and discussion between the scientific community and continued refinement of bioinformatics tools. 

## Data Availability

Not applicable.

## Supplementary Materials

The following are available online at https://www.mdpi.com/1999-4915/13/3/506/s1. Figure S1: Heatmap and dendrogram output of the GRAViTy pipeline as companion information for Figure 2. The clusters corresponding to eukaryotic family level cut-offs were automatically indicated on the figure as part of the pipeline using genus, subfamily and our family information. Figure S2: vContact2 network illustrating families of the viruses of bacteria. Phage genomes were downloaded from GenBank on the 22 February 2021, representing a total of 14,462 sequence records, and reannotated using Prokka via the INfrastructure for a PHAge Reference Database perl script (https://github.com/RyanCook94/inphared.pl (accessed on 5 February 2021)). vContact2 version 0.9.21 was used to cluster phage genomes using the parameters --rel-mode ’Diamond’, db ’None’, pcs-mode MCL and vcs-mode ClusterONE. The resultant network was visualised in Cytoscape v3.8.2 and annotated using a custom python script and Adobe Illustrator. ICTV-ratified families are shown in italic font, while pending proposals for new families are shown in Roman font. Putative phage genomes from metagenomic sequence data held in the Sequence Read Archive are not included, therefore, phages belonging to the classes *Leviviricetes, Tokiviricetes, Laserviricetes* and the proposed new order “*Crassvirales*” are under-represented in this network and their depiction may not be an accurate representation.
