# Supplementary figures and images for: A Roadmap for Genome-Based Phage Taxonomy
Source: Viruses. 2021 Mar 18;13(3):506. doi: 10.3390/v13030506 (PMC8003253; doi:10.3390/v13030506)

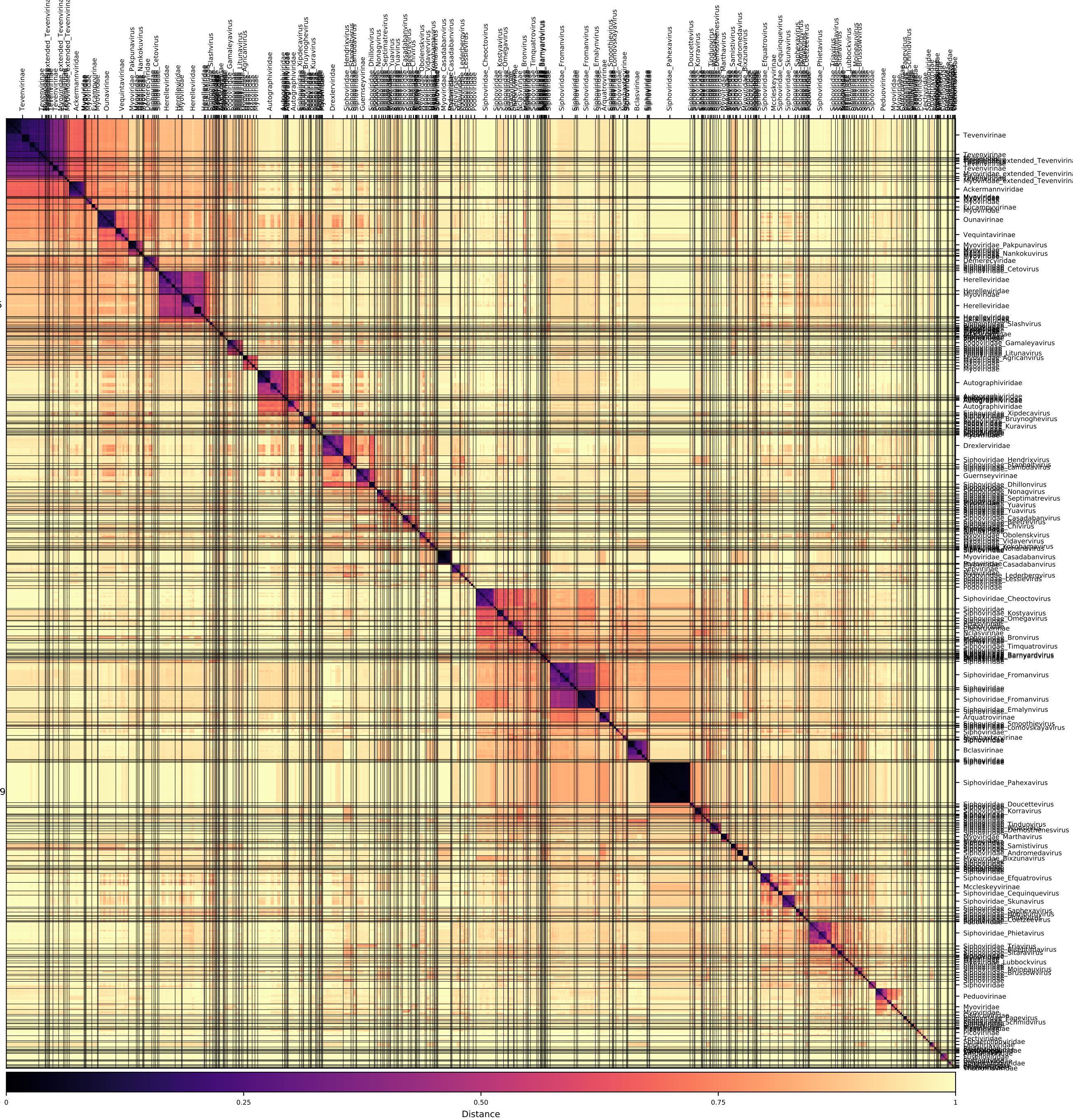

Supplement: Supplementary file 1 [file viruses-13-00506-s001.zip › Supplementary Figure 2.pdf]

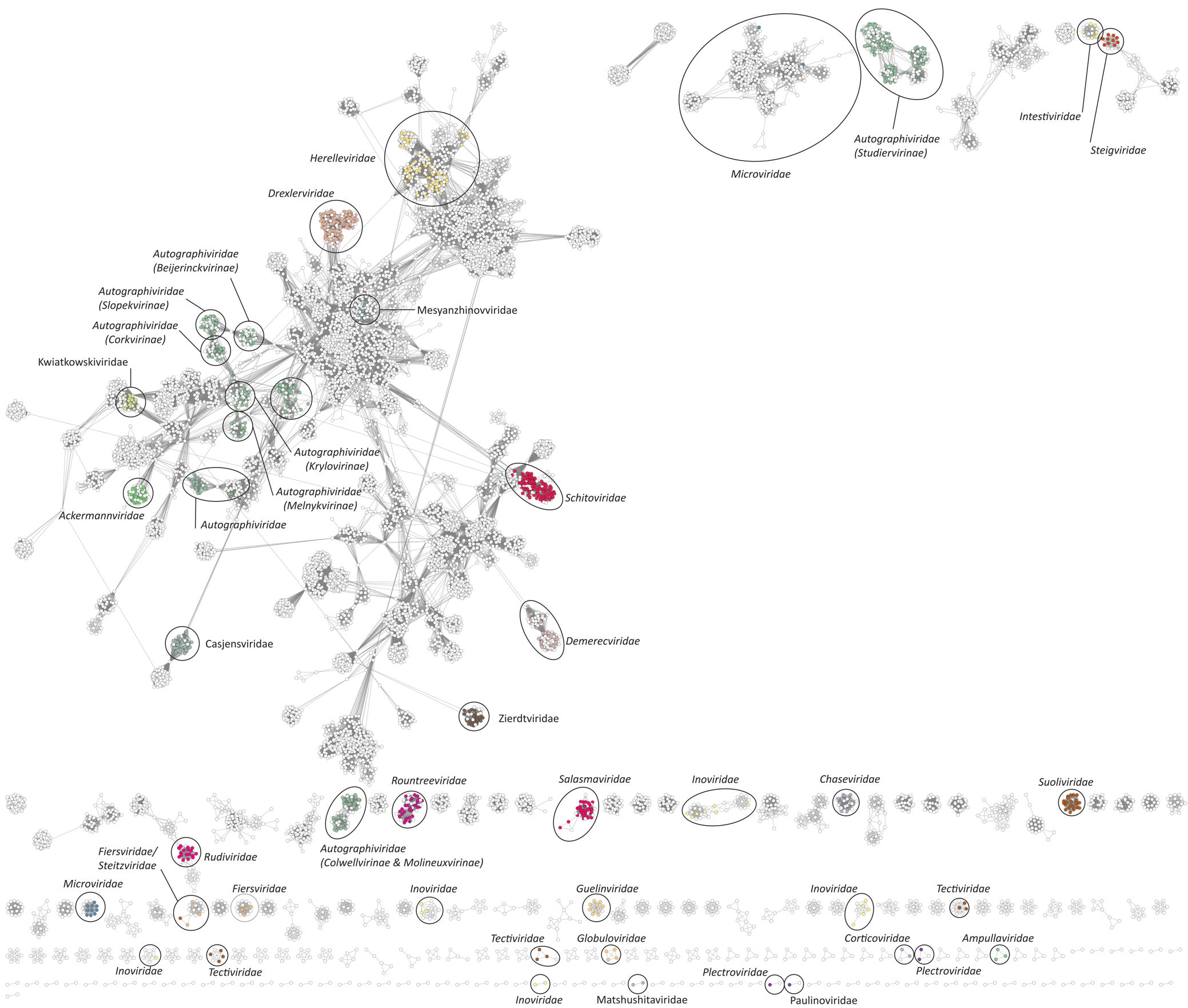

Supplement: Supplementary file 1 [file viruses-13-00506-s001.zip › Supplementary Figure 1.pdf]
